# Ets1 Promotes the Differentiation of Post-Selected iNKT Cells through Regulation of the Expression of Vα14Jα18 T Cell Receptor and PLZF

**DOI:** 10.3390/ijms222212199

**Published:** 2021-11-11

**Authors:** Ya-Ting Chuang, Wan-Chu Chuang, Chih-Chun Liu, Chia-Wei Liu, Yu-Wen Huang, Huang-Yu Yang, I-Cheng Ho, Tzong-Shyuan Tai

**Affiliations:** 1 Department of Medical Research, National Taiwan University Hospital, Taipei 10002, Taiwan; ytchuang1976@gmail.com (Y.-T.C.); monjina36@gmail.com (W.-C.C.); f93449007@ntu.edu.tw (C.-C.L.); ck411030@gmail.com (C.-W.L.); yuwenhuang0719@gmail.com (Y.-W.H.); 2 Kidney Research Center, Department of Nephrology, Chang Gung Memorial Hospital, Taoyuan 33305, Taiwan; hyyang01@gmail.com; 3 College of Medicine, Chang Gung University, Taoyuan 33302, Taiwan; 4 Advanced Immunology Laboratory, Chang Gung Memorial Hospital, Taoyuan 33305, Taiwan; 5 Division of Rheumatology, Inflammation, and Immunity, Department of Medicine, Brigham and Women’s Hospital, 60 Fenwood Road, Boston, MA 02115, USA; iho@bwh.harvard.edu; 6 Harvard Medical School, 60 Fenwood Road, Boston, MA 02115, USA; 7 Department of Medical Research and Development, Chang Gung Memorial Hospital, Taoyuan 33305, Taiwan

**Keywords:** iNKT cells, Ets1, ICOS, PLZF

## Abstract

The transcription factor Ets1 is essential for the development/differentiation of invariant Natural Killer T (iNKT) cells at multiple stages. However, its mechanisms of action and target genes in iNKT cells are still elusive. Here, we show that Ets1 is required for the optimal expression of the Vα14Jα18 T cell receptor (TCR) in post-selected thymic iNKT cells and their immediate differentiation. Ets1 is also critical for maintaining the peripheral homeostasis of iNKT cells, which is a role independent of the expression of the Vα14Jα18 TCR. Genome-wide transcriptomic analyses of post-selected iNKT cells further reveal that Ets1 controls leukocytes activation, proliferation differentiation, and leukocyte-mediated immunity. In addition, Ets1 regulates the expression of ICOS and PLZF in iNKT cells. More importantly, restoring the expression of PLZF and the Vα14Jα18 TCR partially rescues the differentiation of iNKT cells in the absence of Ets1. Taken together, our results establish a detailed molecular picture of how Ets1 regulates the stepwise differentiation of iNKT cells.

## 1. Introduction

Invariant Natural Killer T (iNKT) cells are a unique subset of T cells expressing an invariant TCRα chain (Vα14Jα18 in mouse and Vα24Jα18 in human) [1,2]. These cells differentiate from double-positive (DP) thymocytes and are functionally defined by reactivity to α-galactosylceramide (αGC). Unlike conventional T cells, iNKT cells are selected by CD1d expressing DP thymocytes but not MHC of thymic epithelial cells [3,4]. Upon CD1d-mediated selection, according to the traditional lineage maturation model, immature iNKT cells (CD24^hi^NK1.1−CD44−, stage 0) differentiate into three sequential stages: CD24−NK1.1−CD44− (stage 1), CD24−NK1.1−CD44+ (stage 2), and CD24−NK1.1+CD44+ (stage 3) [5,6]. However, emerging data have supported a lineage differentiation model, in which CD24+ iNKT precursors differentiate directly into three subsets: iNKT1 (T-bet^+^, PLZF^low^) that produces IFN-γ, iNKT2 (GATA3^+^, PLZF^high^) that produces IL-4, and IL-17-producing iNKT17 (RORγt+, PLZF^intermediate^). Generally, iNKT1 cells belong to the stage 3 population, iNKT2 cells belong to the stage 1 and stage 2 populations, and iNKT17 cells belong to the stage 2 population [7].

Mature iNKT cells display features of antigen-experienced T cells and maintain a “poised” effector state at baseline. They are capable of being activated by glycolipid antigens presented by CD1d without priming and secreting a large amount of cytokines, including IFN-γ, IL-4, IL-2, and IL-17A. By producing cytokines in different contexts, iNKT cells can play either a protective role or a pathogenic role in human diseases [8,9,10,11,12,13]. Their differentiation, maturation, homeostasis, and function are tightly regulated by transcription factors, such as Ets1, Egr2, NFkB1, and PLZF [14,15,16,17,18].

Ets1 is the prototype of the ETS family of transcription factor. Ets1 is best known for its role in the modulation of cytokine production and Th cell differentiation [19,20,21,22,23]. It is also required for the proper downregulation of CD4 in CD8 SP thymocytes and regulates the expression of CD127 in thymocytes and peripheral T cells [24,25]. In addition, it critically regulates the differentiation of B cells and group 2 innate lymphoid cells [26,27,28,29]. Its effects on lymphocytes provide an attractive explanation for the autoimmune features seen in Ets1KO mice and its association with lupus in genome-wide association studies [30,31]. Ets1 is expressed at relatively high levels in all stages of iNKT cells. Germline deficiency of Ets1 results in a near absence of iNKT cells, making it challenging to study the roles and mechanisms of action of Ets1 in regulating iNKT cell biology [20,32].

Ets1 contains a Pointed (PNT) domain in its N-terminus. We have previously demonstrated that Ets1 promotes the transition of thymic iNKT cells from the CD24+ stage to CD24− stages through a PNT domain-independent mechanism but inhibits their expression of IL-17 in a PNT domain-dependent manner [32]. Here, we adopted a genetic approach and conducted transcriptomic analyses to further characterize in detail the roles of Ets1 in regulating the selection and differentiation of iNKT cells, and we uncovered several target genes of Ets1 in iNKT cells.

## 2. Results

### 2.1. Regulation of the Immediate Differentiation of Post-Selected iNKT Cells by Ets1

**Figure 1:**
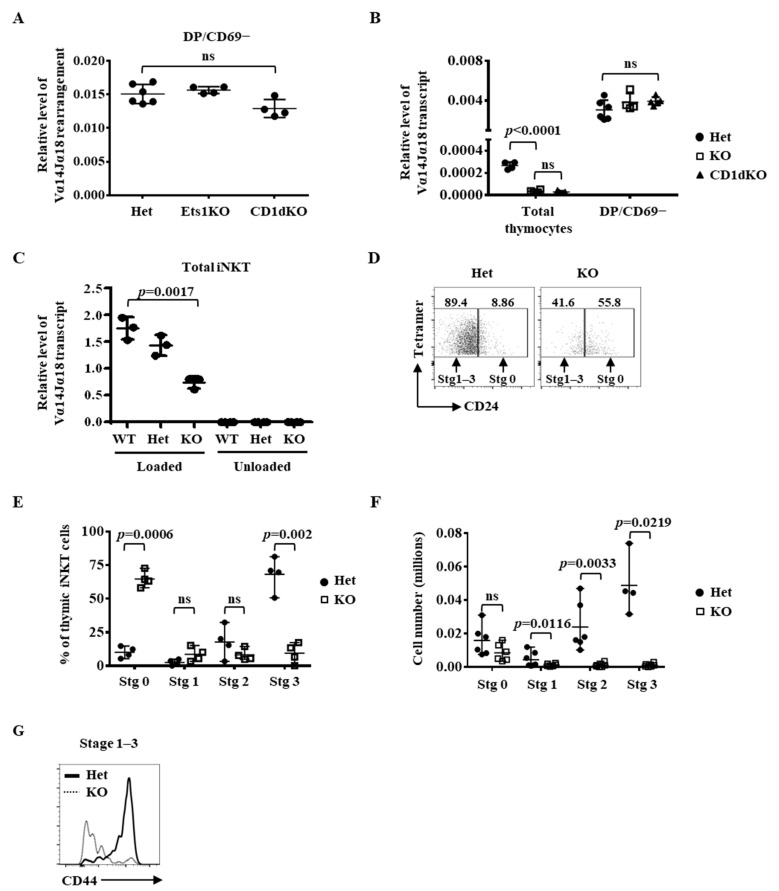
A dispensable role of Ets1 in the expression of Vα14Jα18 TCR in thymic iNKT precursors and their selection. A-C. Genomic DNA (**A**) and cDNA (**B**,**C**) were prepared from total thymocytes (**B**), sorted CD69− DP thymocytes (**A**,**B**), and total iNKT cells (**C**) of indicated genotype and subjected to quantitative PCR using primers corresponding to the Vα14 and Jα18 regions. (**D**–**G**) Thymic iNKT cells were identified with TCR and loaded CD1d tetramer and then subsequently separated into stage 0 and stage 1–3 based on the level of CD24 (**D**). Stage 1–3 cells were further fractionated into stage 1, 2, and 3 based on the expression of NK1.1 and CD44. The percentage of each stage of iNKT cells among total iNKT cells and their absolute numbers were calculated and are shown in E and F, respectively. G. CD44 staining of stage 1–3 iNKT cells is shown. The data shown in Figure 1 are compiled from at least three pairs of mice.

The near absence of iNKT cells in Ets1KO mice suggests that Ets1 regulates the expression of the Vα14Jα18 TCR gene and/or the selection of Vα14Jα18-expressing DP cells. The expression of Vα14Jα18 TCR requires productive re-arrangements between Vα14 and Jα18 followed by transcription of the re-arranged Vα14Jα18. We found that the re-arrangement between Vα14 and Jα18 in the iNKT precursor CD69−DP thymocytes of Ets1KO mice was normal and as efficient as that in those of CD1dKO mice (Figure 1A and Figure S1). Expectedly, there was almost no detectable Vα14Jα18 transcript in the total thymocytes of Ets1KO mice (Figure 1B). However, the level of the Vα14Jα18 transcript in CD69−DP cells was comparable among Ets1 heterozygous (hereafter referred to as Het), Ets1KO, and CD1dKO mice (Figure 1B). Thus, deficiency of Ets1 has little impact on the re-arrangement and expression of Vα14Jα18 TCR in iNKT precursors. Then, we identified and sorted residual thymic “iNKT” cells of Ets1KO mice with CD1d-loaded tetramer and examined the Vα14Jα18 TCR expression. The residual Ets1KO iNKT cells did express Vα14Jα18 TCR transcripts, albeit at a level approximately 50% of that of WT or Het iNKT cells (Figure 1C). The result indicates that the residual cells are bona fide iNKT cells. Approximately 60% of the residual Ets1KO iNKT cells, in contrast to 10% of Ets1Het iNKT cells, were stage 0 (CD24+) cells (Figure 1D,E), but the absolute number of stage 0 Ets1KO thymic iNKT cells was comparable to wild-type counterparts (Figure 1F). There were very few CD24− iNKT cells in Ets1KO thymus (Figure 1D). Nearly all of the Ets1KO CD24− iNKT cells were CD44− cells (Figure 1G), resulting in a near absence of stage 2 and stage 3 cells (Figure 1E,F).

**Figure 2:**
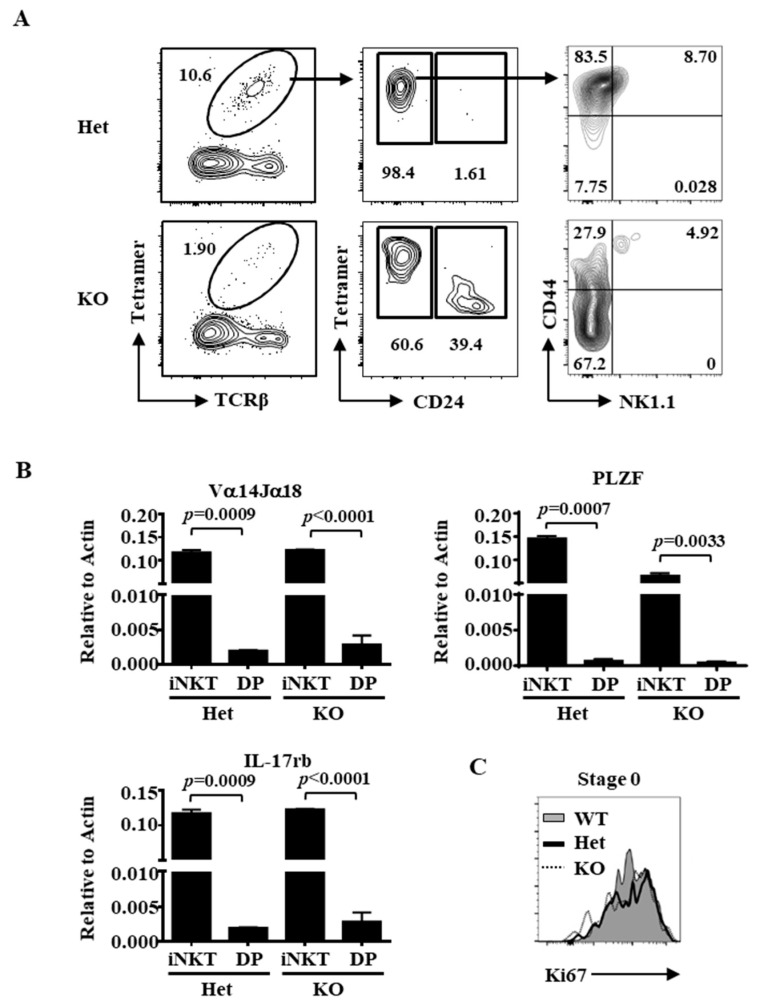
Enrichment of iNKT cells by CD1d-tetramers. (**A**) CD1d-tetramer-enriched iNKT cells from the indicated genotype were identified with TCR and loaded CD1d tetramer (left panels), subsequently separated into stage 0 and stage 1–3 based on the level of CD24, NK1.1, and CD44 (right panels). (**B**) Enriched iNKT cells from the indicated genotype were subjected to quantitative PCR using primers corresponding to the transcripts of Vα14 Jα18 TCR, PLZF, and IL-17rb. (**C**) Ki67 staining of stage 0 iNKT cells is shown.

To further confirm that the residual thymic CD24+ “iNKT” cells in Ets1KO mice were not double positive thymocytes contamination, we enriched iNKT cells with CD1d-αGalCer tetramers magnetic-activated cell sorting (MACS) by more than 100-fold (Figure 2A). Again, approximately 40% of the enriched Ets1 KO iNKT cells, in contrast to only 1.6% of the enriched Ets1Het iNKT cells, were stage 0 cells (Figure 2A). Again, the CD24+ Ets1 KO iNKT cells were mainly CD44−. The expression of iNKT-related genes, such as Vα14Jα18 TCR, PLZF, and IL-17rb, were readily detected in the enriched iNKT cells but not in concomitantly sorted double-positive thymocytes (Figure 2B). These results demonstrate that Ets1 is essential for differentiation of iNKT cells beyond stage 0. This near absence of post-stage 0 cells is not due to impaired proliferation because the Ki67 staining was comparable between WT, Het, and Ets1KO stage 0 iNKT cells (Figure 2C).

### 2.2. Transcriptomic Analyses of Ets1KO Thymic iNKT Cells

**Figure 3:**
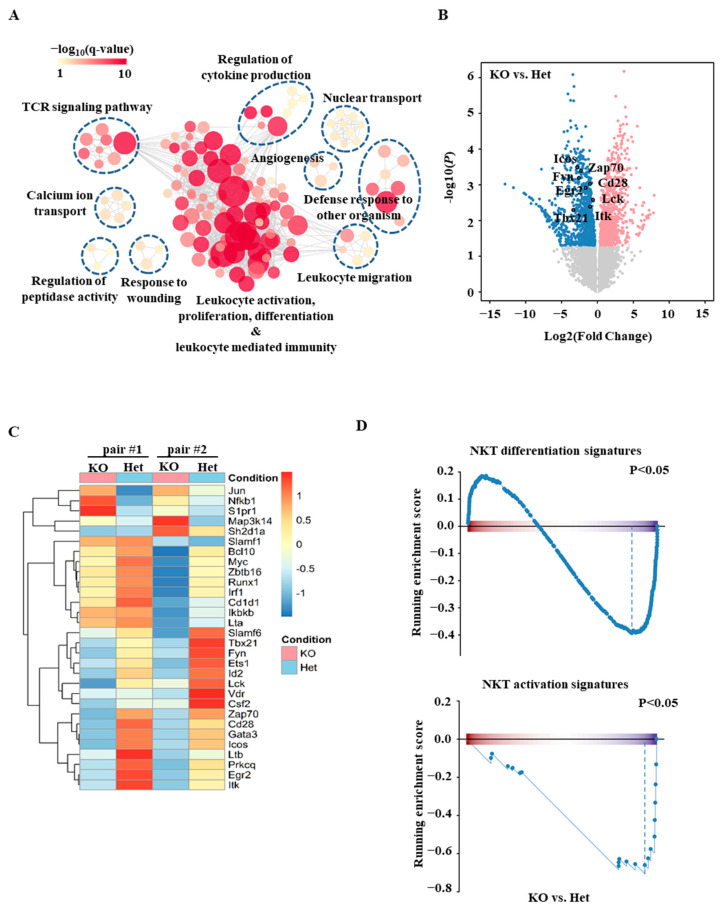
Transcriptomic analyses of Ets1KO iNKT cells. Stage 0 iNKT cells from Het and Ets1KO mice were sorted and subjected to RNA-based next-generation sequencing (RNA-seq). (**A**) The enrichment map shows the significantly suppressed functions in KO compared with Het stage 0 iNKT cells. Nodes are GO gene sets, and edges indicate shared genes between GO gene sets. Node size represents the number of perturbed genes in a given set. (**B**,**C**) Differences in various genes’ expression in Het and KO stage 0 iNKT cells are shown in a volcano plot (**B**) and a heat map (**C**). *p*-value < 0.05 was used to identify differentially expressed genes in the volcano plot. The heat map represents data from two independent RNA-seq. (**D**) The results of GSEA analyses of NKT differentiation signatures (upper panel) and NKT activation signature (lower panel) of Het and KO stage 0 iNKT cells are shown.

To further investigate the mechanism of action of Ets1, we sorted stage 0 iNKT cells from Het and Ets1KO mice and profiled their transcriptomes with RNA-based next-generation sequencing (RNA-seq). Pathway analysis revealed that the most differentially expressed genes between Het and Ets1KO stage 0 iNKT cells are genes related to leukocytes activation, proliferation differentiation, and leukocyte-mediated immunity (Figure 3A). Several of the genes that are involved in leukocyte activation have been shown to regulate the development of iNKT cells. For example, Lck, Fyn, Zap70, and Itk have been shown to regulate the development of iNKT cells, and their expression was attenuated in the absence of Ets1 (Figure 3B,C). To gain further insight into the gene expression signatures of iNKT cells, we performed gene set enrichment analysis (GSEA), which revealed that Ets1KO stage 0 iNKT cells were deprived of the genes related in iNKT cell differentiation and activation compared to the Het stage 0 iNKT cells (Figure 3D and Table S1).

**Figure 4:**
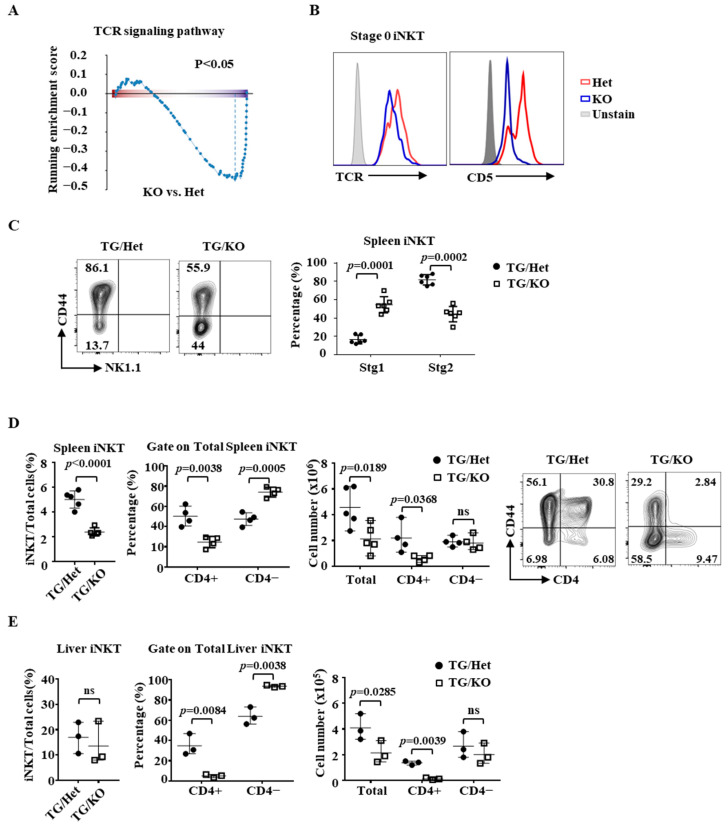
Partial rescue of the differentiation of Ets1KO iNKT cells with a Vα14Jα18 TCR transgene. (**A**) The result of GSEA analysis of the TCR signaling pathway is shown. (**B**) The surface level of TCR and CD5 in thymic iNKT cells of indicated genotypes was compared in the histogram. (**C**) Representative NK1.1/CD44 plots of splenic iNKT cells of indicated genotypes are shown. The percentages of stage 1 and stage 2 splenic iNKT cells from six pairs of TG/KO and TG/Het mice are shown in the right panel. (**D**,**E**) The percentage and absolute numbers of total, CD4+, and CD4− iNKT cells in spleens (**D**) and livers (**E**) of indicated genotypes are shown. Representative CD4/CD44 FACS plots of splenic iNKT cells of indicated genotypes are also shown in (**D**).

GSEA analysis also revealed that the expression of genes in the TCR signaling pathway was reduced in stage 0 Ets1KO iNKT cells (Figure 4A and Table S1), which is a finding that is consistent with the reduced level of Vα14Jα18 TCR transcripts in thymic Ets1KO iNKT cells and attenuated surface expression of TCR and CD5 in stage 0 Ets1KO iNKT cells (Figure 1C and Figure 4B). These results are also in agreement with our published data showing that a CD4 promoter-driven Vα14Jα18 TCR transgene (TG) was able to increase the number of thymic iNKT cells in Ets1KO mice to a level even slightly higher than that of TG/WT mice [32]. However, the increase was mainly in stage 0 and 1 cells, and there was still a marked reduction in the number/percentage of stage 2 thymic iNKT cells of TG/KO mice compared to that of TG/WT mice despite comparable levels of TCR (Figure S2).

A similar but less striking reduction in the ratio between stage 2 and stage 1 iNKT cells was also observed in the peripheral organs, such as spleen, of TG/KO mice compared to that of TG/Het mice (Figure 4C). In addition, TG/KO mice had a substantial reduction in the number of splenic iNKT cells in their spleens (Figure 4D). For example, TG/Het spleens contained approximately 5 million of iNKT cells, which constituted 7% of total splenocytes. However, in TG/KO mice, there were only two million splenic iNKT cells, representing 2% of all splenocytes. This reduction was mainly due to a loss of CD4+ subset mainly in stage 2 (CD44+) but also in stage 1 (CD44−) cells (Figure 4D); the numbers of CD4− splenic iNKT cells were comparable between TG/Het and TG/KO mice. Similar results were obtained with liver iNKT cells (Figure 4E). The levels of TCR in various subsets of TG/KO iNKT cells were similar to those of Het/KO iNKT cells. Taken together, the weaker TCR signaling uncovered in the GSEA analysis can be in part attributed to the attenuated expression of the Vα14Jα18 TCR; however, Ets1 also regulates the differentiation of post-stage 0 thymic iNKT cells and the homeostasis of peripheral iNKT cells independently of the Vα14Jα18 TCR.

### 2.3. Identification of ICOS as a Target Gene of Ets1

**Figure 5:**
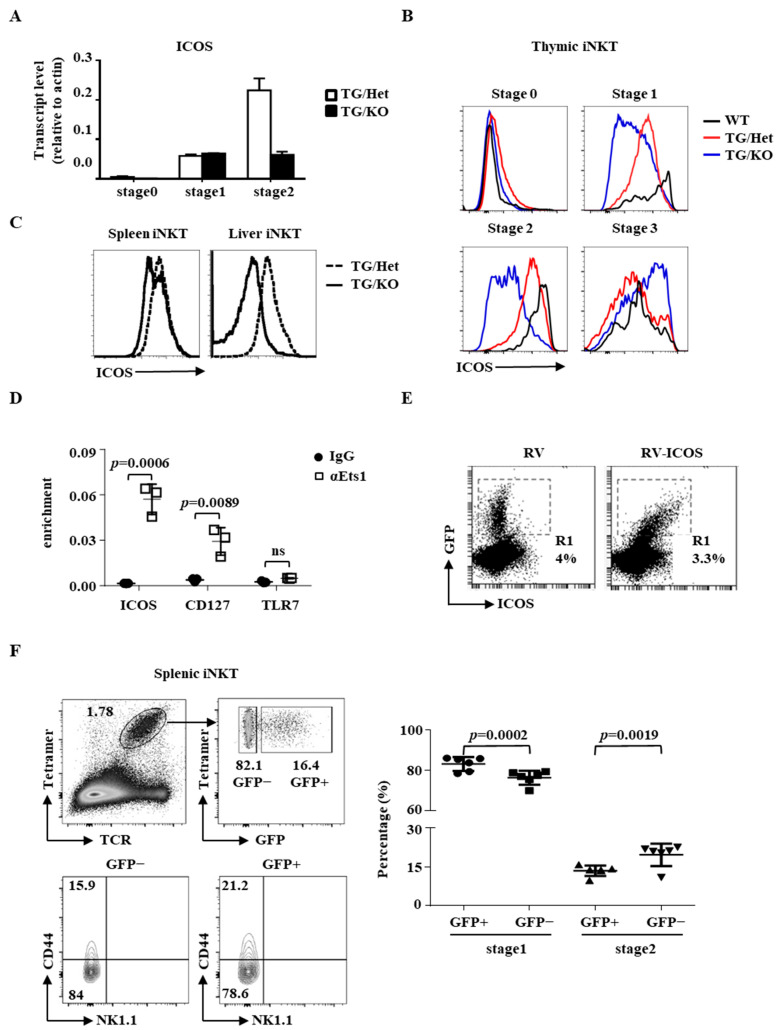
Identification of ICOS as a target gene of Ets1. (**A**) cDNA was prepared from various thymic iNKT subpopulations of indicated genotypes, and the transcript levels of ICOS were quantified with quantitative PCR. (**B**,**C**) The surface levels of ICOS in different stages of thymic iNKT cells (**B**) and peripheral iNKT cells (**C**) are shown. (**D**) Activated Th cells were subjected to ChIP analysis using anti-Ets1 or control IgG. The recruitment of Ets1 to the promoter of ICOS, CD127, and TLR7 was examined with quantitative PCR. (**E**,**F**). Bone marrow cells transduced with RV vector alone (RV) or GFP− ICOS-RV (RV-ICOS) and stained with ICOS. The co-expression of ICOS and GFP are shown in (**E**). Splenic iNKT cells of WT mice reconstituted with GFP−ICOS retrovirus-transduced TG/KO bone marrow cells were identified with TCR/CD1d-tetramer (the upper panel of (**F**)) and divided into GFP+ (transduced) and GFP− (un-transduced) populations (the upper panels of (**F**)). The distribution of stage 1, 2, and 3 GFP+ and GFP− donor iNKT cells was determined by NK1.1 and CD44. Representative FACS plots are shown in the bottom panels of (**F**). Cumulative data from six mice are shown in the right panel of (**F**). Statistical analyses were performed with a paired Student’s *t* test.

The interaction between ICOS on iNKT cells and ICOS ligand on dendritic cells or B cells is critical for the homeostasis and maturation of iNKT cells. While ICOS deficiency has no impact on the homeostasis/maturation of thymic iNKT cells, its deficiency leads to loss of CD4+ iNKT cells in peripheral organs [33], which is a phenotype resembling the peripheral iNKT phenotype of TG/KO mice (Figure 4D,E). We found that the transcript level of ICOS was lower in the stage 2 thymic iNKT cells of TG/KO mice compared to that of TG/Het mice (Figure 5A). The surface level of ICOS in wild-type thymic iNKT cells was low at stage 0, elevated at stage 1, peaked at stage 2, and decreased at stage 3 to a level comparable to that of stage 1 (Figure 5B), which is consistent with its transcript level shown in ImmGen (Figure S3A). The kinetics of ICOS expression was maintained in the iNKT cells of TG/Het mice despite a slightly higher level at stage 0. However, TG/KO iNKT cells were unable to upregulate ICOS during maturation, resulting in a marked reduction in the level of ICOS in stage 1 and stage 2 cells. Attenuated ICOS expression was also detected in peripheral iNKT cells and activated Th cells of TG/KO mice (Figure 5C and Figure S3B). Taken together, the data indicate that Ets1 is required for the proper expression of ICOS at the transcriptional level.

We subsequently analyzed the genomic sequence of the Icos gene with rVista 2.0 (http://rvista.dcode.org, accessed on 28 April 2014) and identified a potential ETS binding site at position −44 to −47 of the Icos gene. Chromatin immunoprecipitation (ChIP) using anti-CD3-stimulated Th cells revealed that Ets1 was recruited to the promoters of ICOS and CD127, which contains a known Ets binding site (Figure 5D). As a control, we detected no recruitment of Ets-1 to the TLR7 promoter, which bears no predicted Ets1 binding site. This result strongly suggests that the ETS site is critical for the activity of the ICOS promoter and supports the notion that ICOS is a direct target of Ets1.

To investigate if ICOS mediates the effect of Ets1 on iNKT cells maturation in vivo, we infected TG/KO bone marrow cells (CD45.2) with GFP/ICOS-expressing bi-cistronic retrovirus. Then, the transduced cells were transplanted into irradiated CD45.1 WT mice, and the transduced CD45.2+ TG/KO iNKT cells in host animals were identified by the co-expression of GFP. Comparing with non-transduced (GFP−) CD45.2+ cells, the GFP+ cells expressed a higher level of ICOS (Figure 5E). Interestingly, the restoration of ICOS reproducibly, albeit modestly, increased the percentage of stage 2 TG/KO iNKT cells in spleens (Figure 5F). A reciprocal decrease in the percentage of stage 1 TG/KO iNKT cells was also observed in the GFP+ population. However, the restoration of ICOS expression had no impact on the ratio between stage 1 and stage 2 thymic iNKT cells (Figure S4A) or the percentage of peripheral CD4+ iNKT cells (Figure S4B).

### 2.4. Partial Rescue of the Differentiation of Ets1KO iNKT Cells by PLZF

**Figure 6:**
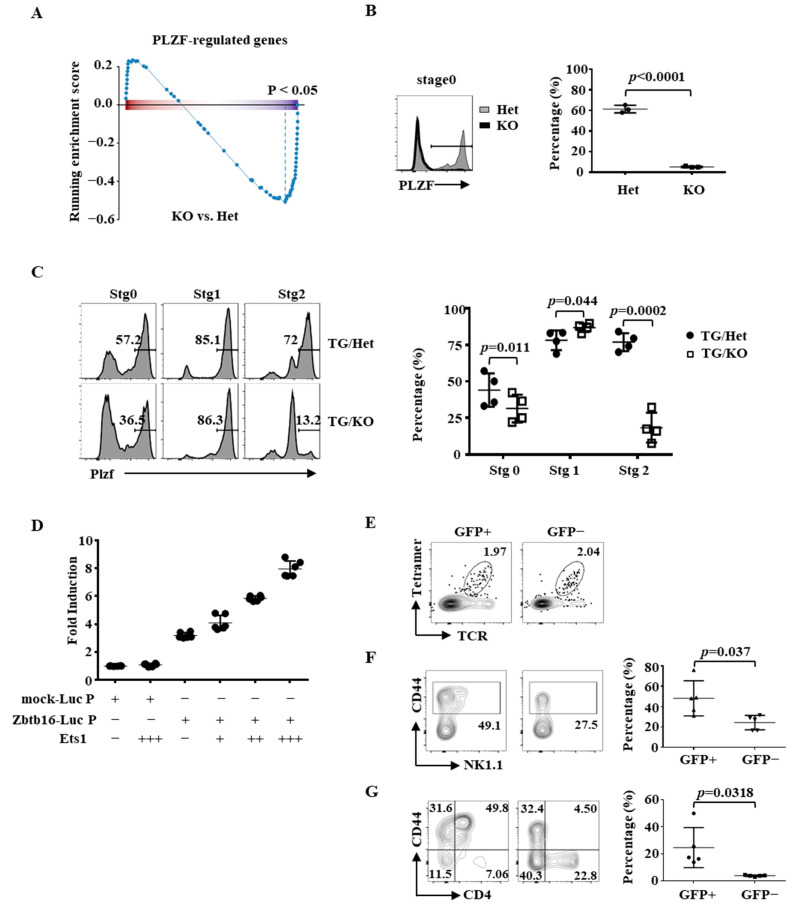
Partial rescue of the differentiation/homeostasis of TG/KO iNKT with PLZF. (**A**) The result of GSEA analyses of Zbtb16 regulated genes is shown. (**B**) PLZF staining of stage 0 iNKT cells of the indicated genotypes is shown. The cumulative results are shown in the right panel. (**C**) PLZF staining of iNKT cells from various stages and indicated genotypes is shown. The cumulative results are shown in the right panel. (**D**) 293 cells were transfected with luciferase reporter only (mock-Luc P) or with Zbtb16 promoter region (Zbtb16-Luc P) and different amounts of Ets1 expression plasmid. The luciferase activity was normalized against internal control obtained from renilla luciferase activity. The normalized activity obtained from mock-Luc was arbitrarily set as 1. The data shown are from three independent experiments. (**E**–**G**) Splenocytes of CD45.1 WT mice reconstituted with GFP-PLZF retrovirus-transduced CD45.2 TG/KO bone marrow cells were separated into CD45.2+GFP+ (transduced) and CD45.2+GFP− (un-transduced) populations and then, iNKT cells were identified with TCR/CD1d-tetramers (**E**). The distribution of stage 1, 2, and 3 as well as the expression of CD4 in GFP+ and GFP− CD45.2 iNKT cells were examined by FACS. Representative NK1.1/CD44 (**F**) and CD44/CD4 (**G**) plots and cumulative data from six mice (the right panels of (**F**,**G**)) are shown. Statistical analyses were performed with a paired Student’s *t*-test.

PLZF is induced immediately after positive selection of iNKT cells and is required for both iNKT cell development and effector functions. Intriguingly, our RNA-seq analysis showed that the level of Zbtb16 (gene encode PLZF) was lower in Ets1KO stage 0 iNKT cells (Figure 3B). GSEA analysis also revealed that the expression of PLZF-regulated genes was reduced in Ets1KO stage 0 iNKT cells (Figure 6A and Table S1). Indeed, flow cytometric analysis revealed that PLZF+ stage 0 thymic iNKT cells seen in Ets1Het mice were missing in Ets1KO mice (Figure 6B), confirming the RNA-seq data. The impaired expression of PLZF was still observed in the stage 0 TG/KO thymic iNKT cells; however, it was not seen in stage 1 cells, but it re-appeared in stage 2 cells (Figure 6C). These results suggest that the dependence on Ets1 for the expression of PLZF is stage-specific and cannot be fully mitigated by the expression of the Vα14Jα18 TCR transgene.

PLZF is dispensable for the transition from CD24+ stage 0 to CD24− post-stage 0 iNKT cells; however, PLZF deficiency results in a marked reduction in the number/percentage of stage 2 and 3 thymic iNKT cells and CD4+ splenic iNKT [34]. These features resemble the phenotype of Ets1 TG/KO mice (Figure S2 and Figure 4C,D), raising the possibility that Ets1 controls iNKT cells differentiation through PLZF. We found that Ets1 transactivated a ≈2 kb Zbtb16 promoter in a dose-dependent manner (Figure 6D), indicating that PLZF is directly regulated by Ets1. Then, we infected TG/KO bone marrow cells (CD45.2) with GFP/PLZF-expressing bi-cistronic retrovirus. The transduced cells were subsequently transplanted into irradiated CD45.1 WT mice, and the transduced KO iNKT cells in host animals were identified by the co-expression of CD45.2 and GFP (Figure 6E and Figure S5). We found that retroviral PLZF had little impact on the distribution of various stages of thymic iNKT cells (Figure S5). However, retroviral PLZF shifted the distribution of splenic iNKT cells toward stage 2 and 3 (Figure 6F). There was a higher percentage of CD44+ cells among CD45.2+GFP+ iNKT cells compared to that of the CD45.2+GFP− iNKT population in spleens. A fraction of the GFP+CD44+ splenic iNKT cells also expressed NK1.1, which is a feature of stage 3 cells. Interestingly, those CD44+NK1.1+ cells also expressed a low level of CD4 (Figure 6G and Figure S6), resulting in the appearance of CD44+CD4+ cells, which were virtually absent in TG/KO spleens (Figure 4D), only in the GPF+ population. Thus, Ets1 directly transactivates PLZF, and restoring the expression of PLZF partially normalizes the differentiation of TG/KO iNKT cells in the spleen.

## 3. Discussion

The approaches described above have enabled us for the first time to characterize in detail the role and mechanism of action of Ets1 in iNKT cells. Our data demonstrate that Ets1 is dispensable for the development of stage 0 iNKT cells. However, it has a broad and profound impact on the transcriptome of the development of iNKT cells and therefore is essential for their differentiation beyond stage 0. Such a broad impact on the transcriptome very likely explains why its action cannot be fully recapitulated by the restoration of the Vα14Jα18 TCR and/or PLZF, which are two target genes of Ets1.

Our finding is consistent with the observation that Ets1 binds to the enhancer downstream to the constant region of TCRα gene [35]. However, the dependence on Ets1 for the expression of TCR appears to be unique to iNKT cells because the level of TCR in Ets1KO conventional T cells is normal [36]. It is also clear that restoring the expression of TCR or PLZF is insufficient to fully rescue the differentiation of Ets1KO iNKT cells. Indeed, our RNA-seq analysis showed that a deficiency of Ets1 affects the expression of many genes, including Lck, Fyn, Zap70, and Itk, that are critical for the normal differentiation of iNKT cells. The data indicate that Ets1 controls iNKT cell development through multiple targets, and the restoration of one or two of the targets led to only partial rescue of the defect caused by Ets1 deficiency. It remains to be determined whether the other target genes are directly or indirectly regulated by Ets1.

PLZF expression is regulated by TCR signaling [37]. The defect of PLZF expression we observed in Ets1-deficient mice may be due to the downregulation of TCR expression. However, the effect of Ets1 on the expression of PLZF in iNKT cells is complicated and appears to be stage-dependent. For example, Ets1 is important for the optimal expression of PLZF in stage 0 iNKT cells of KO or TG/KO mice but is dispensable in stage 1 iNKT cells of TG/KO mice. Recently, it was discovered that the expression of PLZF in iNKT cells is dictated by an intronic enhancer [38]. The deletion of a subregion (+21/23) within the enhancer markedly reduced the level of PLZF in stage 0 but not stage 1 or stage 2 iNKT cells. It is possible that the binding of Ets1 to the +21/23 region is essential for the upregulation of PLZF in stage 0 iNKT cells, but other subregions within the enhancer are required for Ets1 to sustain the expression of PLZF in more mature iNKT cells.

While Ets1 regulates the expression of PLZF and ICOS, the phenotype of iNKT cells caused by Ets1 deficiency is identical to that caused by deficiency in PLZF or ICOS. Thus, it is no surprise that retroviral PLZF or ICOS only partly or fail to rescue the maturation/homeostasis of Ets1KO iNKT cells. Our gene expression analyses also indicate that Ets1 acts independently of other known iNKT regulators, such as Egr2 and NF-kB [1]. Instead, Ets1 directly or indirectly regulates the expression of several genes involved in leukocyte activation during stage 0 and activates a transcriptional program responsible for the differentiation of stage 2 and 3 iNKT cells. Although the functional role of many putative Ets1 targets in iNKT cells identified in our analyses has yet to be studied, we strongly argue that the profound maturation defects seen in the absence of Ets1 are the result of impaired expression of many but not just one of its putative target genes.

Intriguingly, one of the target genes thus identified is CD127, which is a known target of Ets1 in Th cells [25]. A paper indicates that the T cell-specific deficiency of CD127 leads to an impaired proliferation of iNKT cells [39]; a reduction in number is seen in all stages of thymic iNKT cells. However, the phenotype of TG/KO thymic iNKT cells is much more profound than and qualitatively different from that of CD127-deficient iNKT cells. Therefore, it is very unlikely that the impaired CD127 expression is the cause of the near absence of iNKT cells in Ets1KO mice. The role of CD127 in the function of iNKT cells has yet to be characterized; it is still unclear whether the attenuated expression of CD127 can explain the cytokine defects caused by Ets1 deficiency [32].

We have previously shown that the T cell-specific deletion of the PNT domain of Ets1 leads to an increase in the percentage/number of the CD4+ subset of thymic and splenic iNKT cells [32]. This observation is seemingly in conflict with the reduction in the CD4+ subset of peripheral iNKT cells seen in TG/KO mice. The cause of this discrepancy is still unclear and may be attributed to an unexpected and partial rescue effect of the Vα14Jα18 TCR transgene in the absence of Ets1. Alternatively, the functional balance between the PNT and non-PNT domains of Ets1 may critically determine the ratio between CD4+ and CD4- subsets of iNKT cells. The generation of additional genetically engineered mice examining various functional domains of Ets1 in the absence of the Vα14Jα18 TCR transgene may provide clues to this puzzle.

## 4. Materials and Methods

### 4.1. Mice

Ets1KO mice of N5 CH57BL/6 genetic background have been described previously [20]. CD1d-deficient mice were purchased from The Jackson Laboratory (Bar Harbor, ME, USA). Heterozygous or wild-type littermates were used as controls. Vα14Jα18 TCR transgenic mice are courtesy of Dr. Albert Bendelac and were crossed to Ets1KO mice to generated TG/KO mice [40]. All animals were housed under specific pathogen-free conditions, and experiments were performed in accordance with the institutional guidelines for animal care at the College of medicine, National Taiwan University under approved protocols.

### 4.2. Real-Time PCR

Total RNA was extracted with TRIzol reagent, and cDNA was synthesized using the PureLink RNA Mini Kit (Life Technologies, Bedford, MA, USA) or the QuantiTect Reverse Transcription Kit (QIAGEN, Valencia, CA, USA). Gene expression was determined by Brilliant II SYBR Green Kit and Mx3005P QPCR System (Agilent Technologies, Santa Clara, CA, USA) according to the manufacturer’s instructions. All experiments were done in duplicate.

### 4.3. FACS Analysis and Antibodies

The following clones of antibody were purchased from Biolegend (San Diego, CA, USA) and used for cell surface staining: CD4 (RM4-5), TCRβ (H57-597), NK1.1 (PK136), CD44 (IM7), CD24 (M1/69), CD127 (A7R34), CD69 (H1.2F3), CD25 (PC61), CD5 (53-7.3). For nuclear protein staining, cells were fixated and permeabilized using the FOXP3 transcription factor staining kit (Thermo Fisher Scientific, Waltham, MA, USA), according to the manufacturer’s instructions. The following antibodies were used: Ki67 (16A8) and PLZF (Mags.21F7) were purchased from Biolegend. Flow cytometry was performed on a FACSCanto II or FACSLSRII (BD Bioscience, San Jose, CA, USA) and analyzed with FlowJo software (version 10.8.1, BD Bioscience, San Jose, CA, USA). CD1d tetramer, either loaded or unloaded with αGC, was obtained from NIH Tetramer Core Facility (Atlanta, GA, USA).

### 4.4. iNKT Cell Enrichment

Thymus were processed to single cell suspension in 2% FBS, 2 mM EDTA in PBS (1 × 10^7^ cells/100 µL), and incubated with 0.1 µL phycoerythrin (PE)-conjugated CD1d-Tetramers for 15 min at 4 °C. Excess antibodies were washed out. Then, anti-PE Microbeads (130-048-801, Miltenyi Biotec, Bergisch Gladbach, Germany) were added to cell suspension at a ratio of 20 µL beads and 80 µL buffer per 1 × 10^7^ cells, and then, they were incubated for 15 min at 4 °C. Unbound beads were washed out, and cell pellets were resuspended to a concentration of 1 × 10^7^ cells/50 µL. CD1d+ cells were selected by magnetic separation using LS Columns (130-042-401, Miltenyi Biotec, Bergisch Gladbach, Germany). The cell suspension was applied onto the LS Column. Unlabeled cells were washed out by pipetting 3 mL of buffer onto the LS column thrice. The LS Column was removed from the separator, and 5 mL of buffer were pipetted onto the LS Column. The magnetically labeled cells were immediately flushed out. For staining of CD1d+ cells, the following antibodies were used: Alexa Fluor 700 anti-TCR-β (H57-597), Brilliant Violet 510 anti-CD24 (M1/69), Brilliant Violet 605 anti-CD44 (IM7), and Brilliant Violet 785 anti-NK1.1 (PK136).

### 4.5. RNA Sequencing of Thymic Stage 0 NKT Cells

Thymocytes of Ets1 Het and KO mice were stained with TCRβ, PBS57-loaded CD1d tetramer, CD24, CD44, and NK1.1 to sort stage 0 NKT cells on FACSAriaIII (BD Bioscience, San Jose, CA, USA). The sorted cells were directly collected into the lysis buffer and converted to cDNA and pre-amplified using the SMART-Seq^®^ v4 Ultra^®^ Low Input RNA Kit for Sequencing (Clontech Laboratories, Mountain View, CA, USA). A sequencing library was generated from 150 pg amplified cDNA using the Illumina Nextera XT kit (Illumina, San Diego, CA, USA). Each sample was given a unique sample barcode during this step. All samples were pooled and sequenced on the NextSeq500 system to obtain 75 cycles of single-end reads.

Raw reads were aligned to the mouse reference genome GRCm38, and then, gene-level quantification was performed by STAR software. Read counts were normalized by the trimmed mean of M-values (TMM) method implemented in the R package edgeR(version 3.36.0). Differential expression analyses were done using the R package NOISeq(version 2.38.0) with the default settings. The genes with the probability of differential expression > 0.8 and fold-change > 1.5 were defined as differentially expressed genes. Gene Ontology (GO) enrichment analysis was performed with the R package clusterProfiler (version 4.2.0). Significantly enriched GO terms (*p*-value < 0.05) were visualized as an enrichmentMap with cutoff > 0.5 Jaccard-overlap combination score.

### 4.6. Plasmid, Transfection, and Luciferase Assay

The Ets1 expression construct has been described [20]. The 3 kb region of the mouse PLZF promoter was amplified from the genomic DNA of EL4 cells by PCR using primers 5′-GGGGTACCAAGGAAAGCAGTGTCGTGGC-3′ and 5′-CCCGCTCGAGATCGGATACCAGTCCCCTCC-3′ and cloned into a pGL3-Basic luciferase plasmid vector. The −288 to −1 region of the mouse ICOS promoter was amplified from the genomic DNA of EL4 cells by PCR using primers 5′-GGGGTACCTTCATACATGCATGCA-3′ and 5′-CCCAAGCTTGCTCAAAAGTGTCAG-3′ and cloned into a pGL3-Basic luciferase vector. Site-specific mutagenesis of potential Ets binding sites was performed using the Q5 Site-directed Mutagenesis Kit (NEB, Ipswich, MA, USA) following the manufacturer’s instructions. The following primers were used for mutagenesis: 5′-ACAAACCCAACTAGCTCTCCAGAAAAC-3′ and 5′-GGAGGTTGATGTGGTGTC-3′. In all luciferase assays, cells were transfected with 10 μg of luciferase reporter, 5 μg of pcDNA3 vector, and 5 μg pTK-Renilla by BIORAD GENE PULSER II at 280 V and 0.975 F. Luciferase activity was determined in duplicate with Dual-Luciferase^®^ Reporter Assay System (Promega, Madison, WI, USA). The firefly luciferase activity obtained from each sample was normalized against the Renilla luciferase activity from the same sample.

### 4.7. Preparation of Splenic and Liver iNKT Cells

Spleen were harvested and minced. Then, red blood cells were lysed by RBC lysis buffer. Splenocytes were filtered through a 70 uM mesh and used for the staining of iNKT cells. Livers were perfused with PBS, minced, and filtered through a 70 μM mesh. A mixture of histopaque (histopague-1077:histopaque-1119 = 1:0.44) (Sigma-Aldrich, St. Louis, MO, USA) was gently layered on the liver suspension. After the centrifugation at 2000 rpm for 25 min, enriched iNKT cells populations were recovered from the interface and typically contained 10% to 20% iNKT cells.

### 4.8. Bone Marrow Transfer for PLZF and ICOS Restoration

Donor Ets1 KO mice (CD45.2) were received 120 mg/kg 5-FU 4 days prior to bone marrow harvests. Sca-1+ bone marrow cells were stimulated with mIL-3 (20 ng/mL), hTOP (50 ng/mL), mSCF (50 ng/mL), and Flt3-L (50 ng/mL), and then spin infected with GFP/PLZF or GFP/ICOS-expressing bi-cistronic retrovirus twice on day 2 and day 3. Then, 48 h after final transduction, viable cells were harvested and resuspended in sterile PBS at 1 × 10^7^ cells/mL. Recipient congenic CD45.1 mice were irradiated (900 Gy) and received 1 × 10^6^ donor cells injected by intravenous administration. Analysis of GFP positive CD45.2 positive cells was performed by flow analysis at 8 weeks post-transplant. Analysis of iNKT cells was determined by flow analysis using antibodies including CD45.1, CD45.2, PBS57-loaded CD1d tetramer, TCRβ, CD44, NK1.1, and CD4 (BD Bioscience, San Jose, CA).

### 4.9. Statistical Analysis

Statistical analyses were performed with Student’s *t*-test unless indicated otherwise; ns stands for not significant.

## Data Availability

Data will be provided upon reasonable request.

## Supplementary Materials

The following are available online at https://www.mdpi.com/article/10.3390/ijms222212199/s1.
