# Supplementary material for: Ets1 Promotes the Differentiation of Post-Selected iNKT Cells through Regulation of the Expression of Vα14Jα18 T Cell Receptor and PLZF
Source: Int J Mol Sci. 2021 Nov 11;22(22):12199. doi: 10.3390/ijms222212199 (PMC8621504; doi:10.3390/ijms222212199)
Supplement: Supplementary file 1 [file ijms-22-12199-s001.zip › ijms-1405796-supplementary.pptx]

## Slide 1
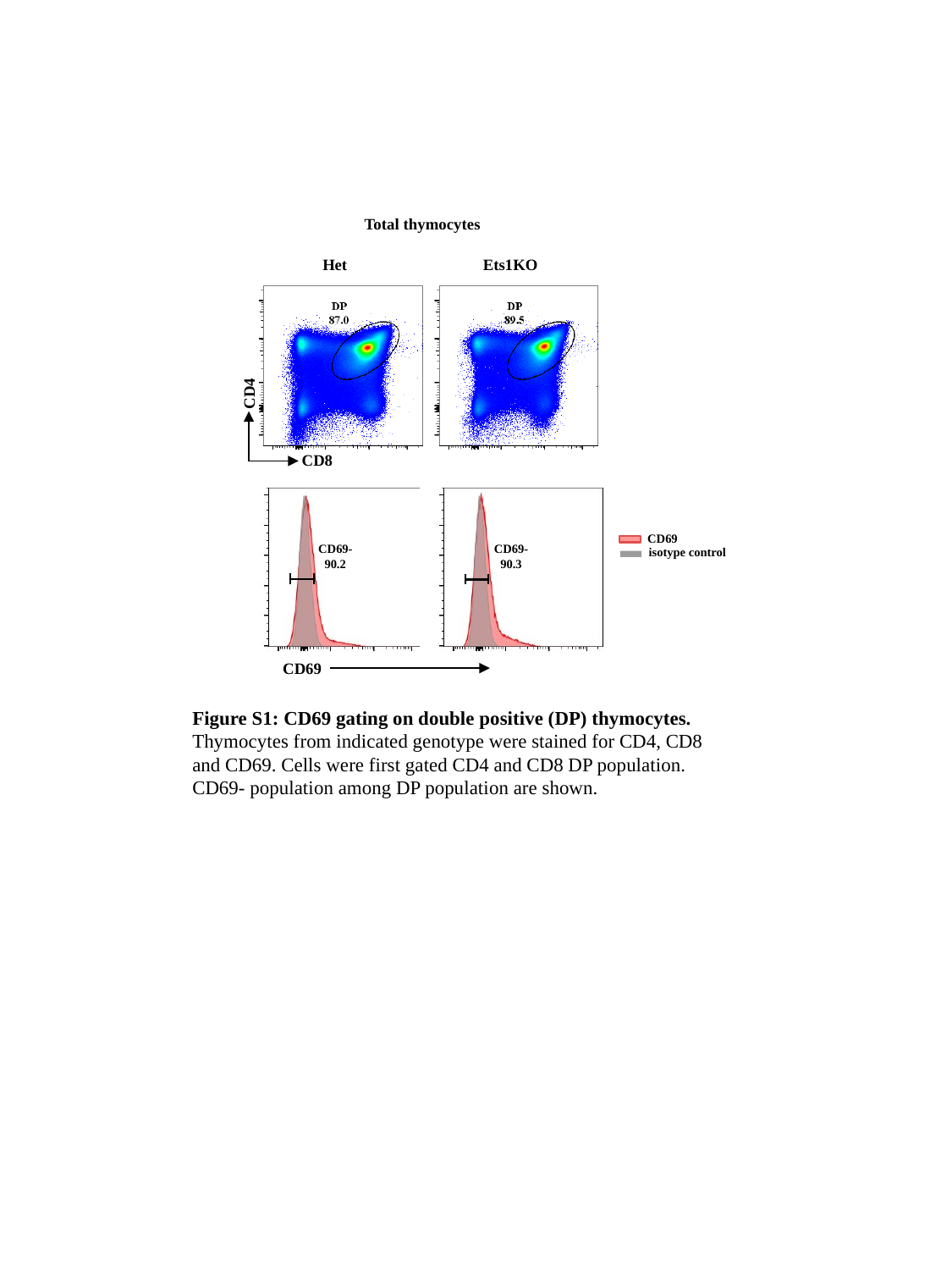

Total thymocytes
Het
Ets1KO
CD4
CD8
CD69
CD69-
90.2
CD69-
90.3
isotype control
CD69
Figure S1: CD69 gating on double positive (DP) thymocytes. Thymocytes from indicated genotype were stained for CD4, CD8 and CD69. Cells were first gated CD4 and CD8 DP population. CD69- population among DP population are shown.

## Slide 2
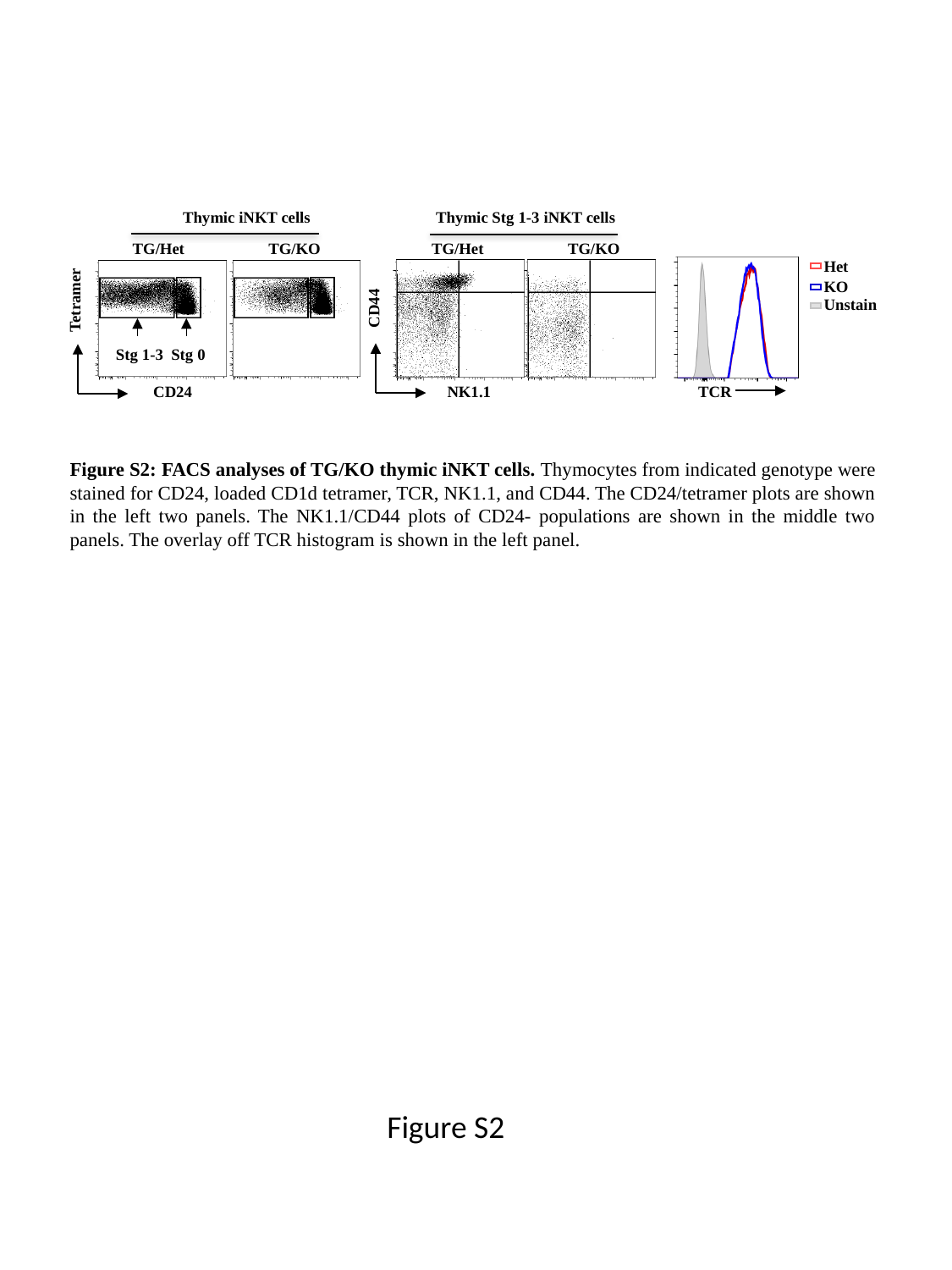

Thymic iNKT cells
Thymic Stg 1-3 iNKT cells
TG/Het
TG/KO
TG/Het
TG/KO
Het
KO
Unstain
Tetramer
CD44
Stg 1-3
Stg 0
NK1.1
TCR
CD24
Figure S2: FACS analyses of TG/KO thymic iNKT cells. Thymocytes from indicated genotype were stained for CD24, loaded CD1d tetramer, TCR, NK1.1, and CD44. The CD24/tetramer plots are shown in the left two panels. The NK1.1/CD44 plots of CD24- populations are shown in the middle two panels. The overlay off TCR histogram is shown in the left panel.
Figure S2

## Slide 3
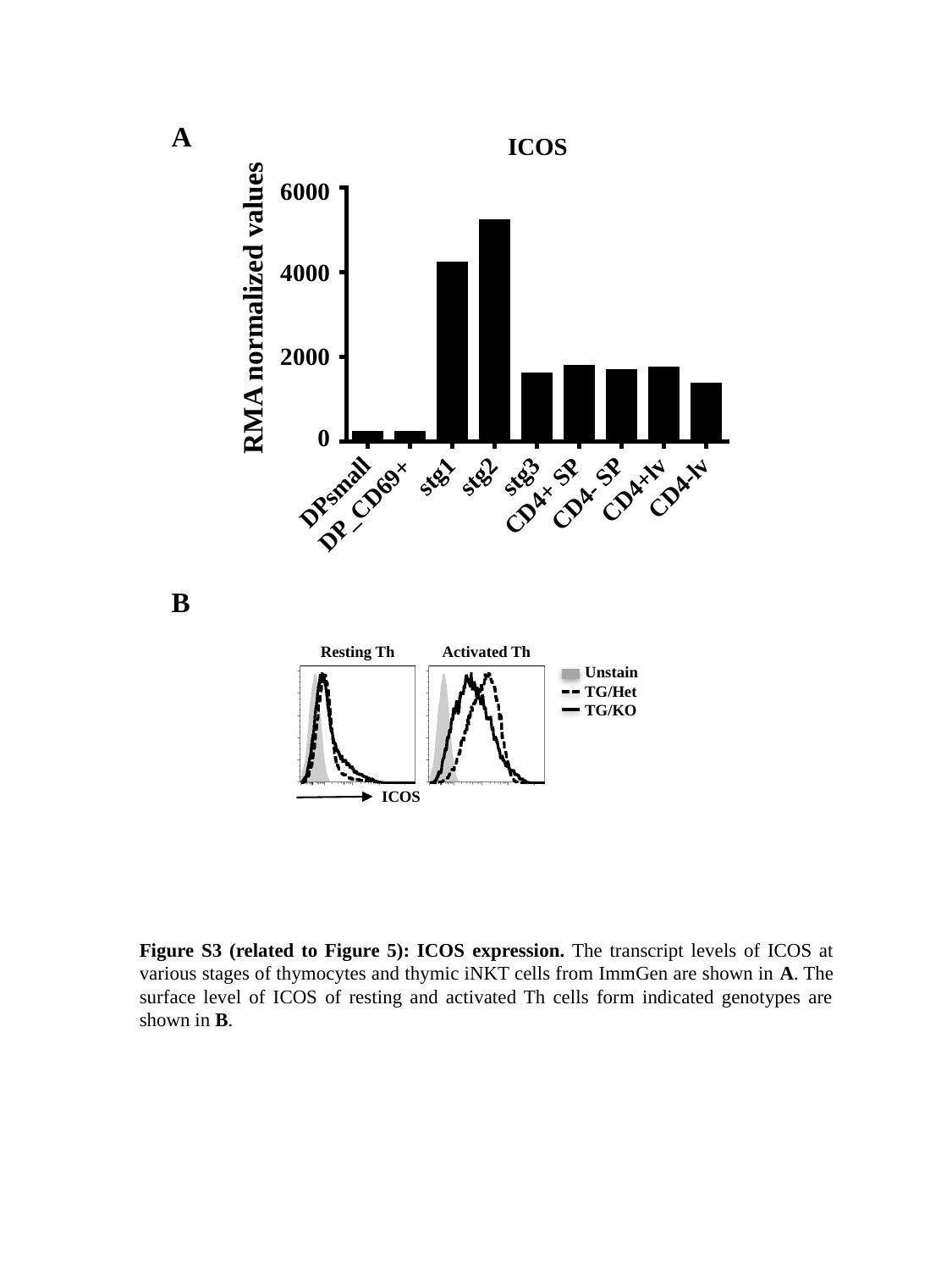

A
6000
4000
RMA normalized values
2000
0
B
Resting Th Activated Th
Unstain
TG/Het
TG/KO
ICOS
Figure S3 (related to Figure 5): ICOS expression. The transcript levels of ICOS at various stages of thymocytes and thymic iNKT cells from ImmGen are shown in A. The surface level of ICOS of resting and activated Th cells form indicated genotypes are shown in B.

## Slide 4
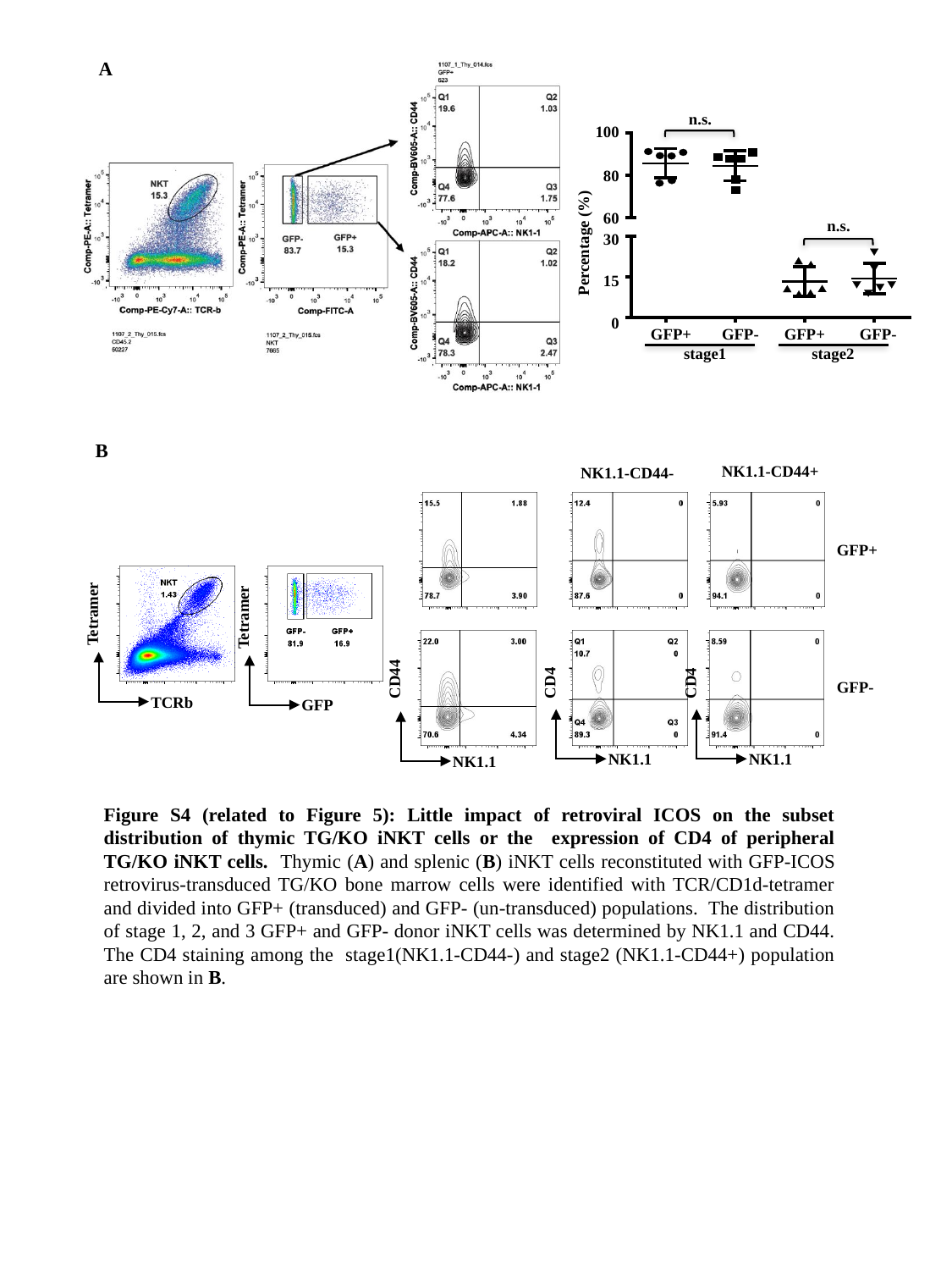

A
n.s.
100
80
60
n.s.
30
15
0
 GFP-
 GFP+
 GFP-
 GFP+
 stage1
stage2
B
NK1.1-CD44+
NK1.1-CD44-
GFP+
Tetramer
TCRb
Tetramer
GFP
CD44
NK1.1
CD4
NK1.1
CD4
NK1.1
GFP-
Percentage (%)
Figure S4 (related to Figure 5): Little impact of retroviral ICOS on the subset distribution of thymic TG/KO iNKT cells or the expression of CD4 of peripheral TG/KO iNKT cells. Thymic (A) and splenic (B) iNKT cells reconstituted with GFP-ICOS retrovirus-transduced TG/KO bone marrow cells were identified with TCR/CD1d-tetramer and divided into GFP+ (transduced) and GFP- (un-transduced) populations. The distribution of stage 1, 2, and 3 GFP+ and GFP- donor iNKT cells was determined by NK1.1 and CD44. The CD4 staining among the stage1(NK1.1-CD44-) and stage2 (NK1.1-CD44+) population are shown in B.

## Slide 5
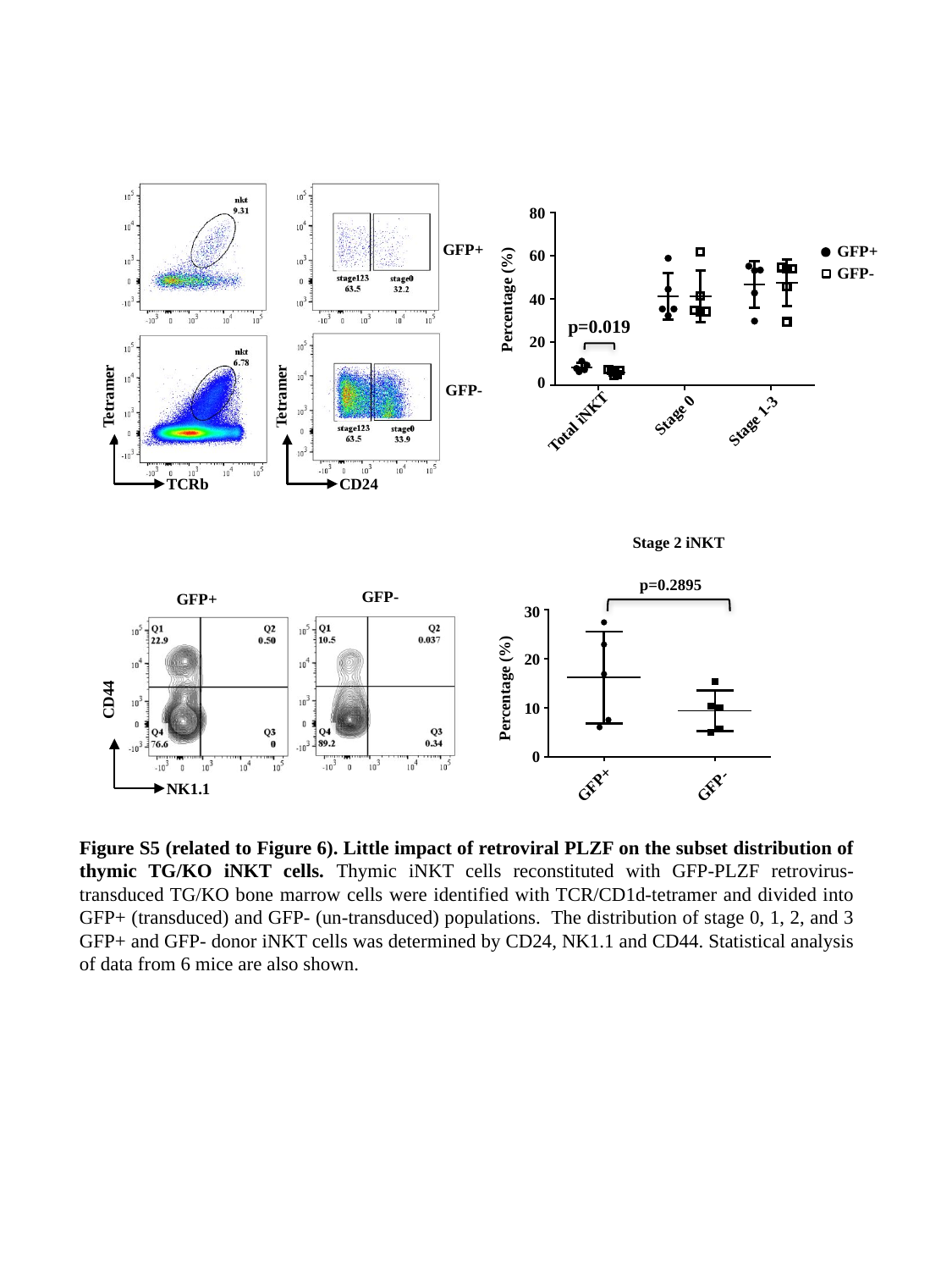

GFP+
GFP-
p=0.019
Stage 0
Stage 1-3
Total iNKT
80
Percentage (%)
GFP+
60
40
20
Tetramer
TCRb
Tetramer
CD24
0
GFP-
Stage 2 iNKT
p=0.2895
GFP-
GFP+
30
Percentage (%)
20
CD44
NK1.1
10
0
GFP+
GFP-
Figure S5 (related to Figure 6). Little impact of retroviral PLZF on the subset distribution of thymic TG/KO iNKT cells. Thymic iNKT cells reconstituted with GFP-PLZF retrovirus-transduced TG/KO bone marrow cells were identified with TCR/CD1d-tetramer and divided into GFP+ (transduced) and GFP- (un-transduced) populations. The distribution of stage 0, 1, 2, and 3 GFP+ and GFP- donor iNKT cells was determined by CD24, NK1.1 and CD44. Statistical analysis of data from 6 mice are also shown.

## Slide 6
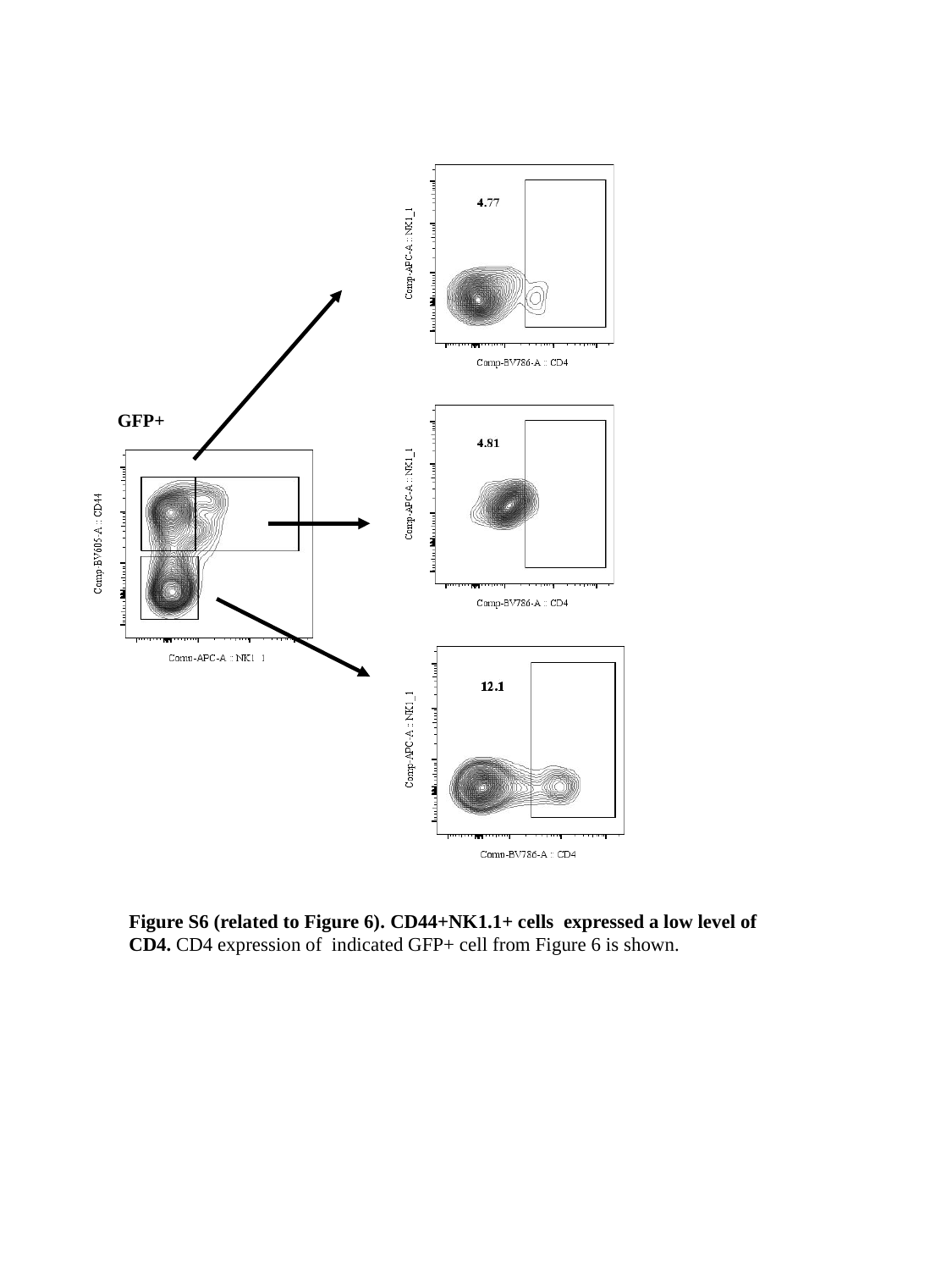

GFP+
Figure S6 (related to Figure 6). CD44+NK1.1+ cells expressed a low level of CD4. CD4 expression of indicated GFP+ cell from Figure 6 is shown.

## Slide 7
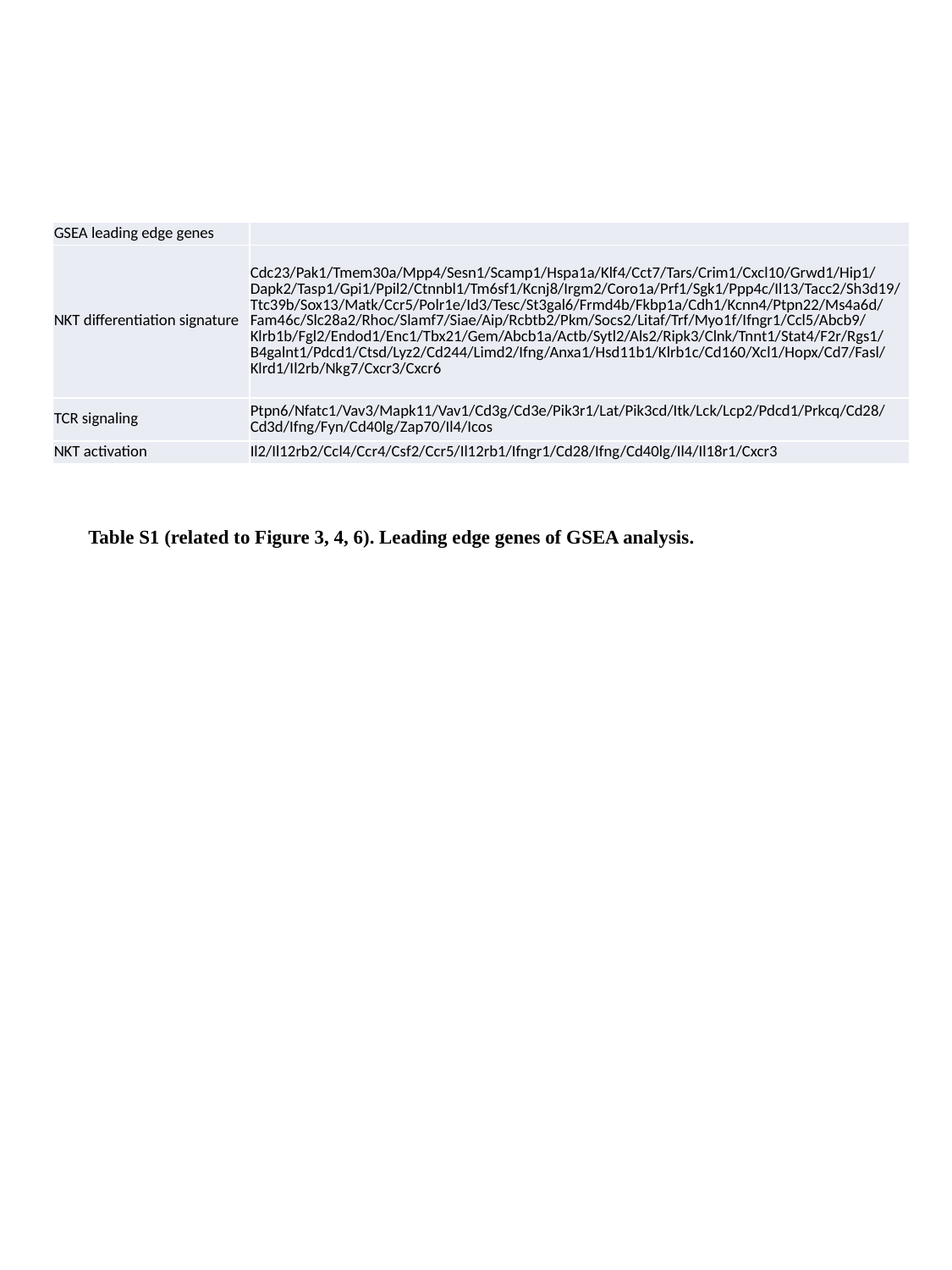

| GSEA leading edge genes | |
| --- | --- |
| NKT differentiation signature | Cdc23/Pak1/Tmem30a/Mpp4/Sesn1/Scamp1/Hspa1a/Klf4/Cct7/Tars/Crim1/Cxcl10/Grwd1/Hip1/Dapk2/Tasp1/Gpi1/Ppil2/Ctnnbl1/Tm6sf1/Kcnj8/Irgm2/Coro1a/Prf1/Sgk1/Ppp4c/Il13/Tacc2/Sh3d19/Ttc39b/Sox13/Matk/Ccr5/Polr1e/Id3/Tesc/St3gal6/Frmd4b/Fkbp1a/Cdh1/Kcnn4/Ptpn22/Ms4a6d/Fam46c/Slc28a2/Rhoc/Slamf7/Siae/Aip/Rcbtb2/Pkm/Socs2/Litaf/Trf/Myo1f/Ifngr1/Ccl5/Abcb9/Klrb1b/Fgl2/Endod1/Enc1/Tbx21/Gem/Abcb1a/Actb/Sytl2/Als2/Ripk3/Clnk/Tnnt1/Stat4/F2r/Rgs1/B4galnt1/Pdcd1/Ctsd/Lyz2/Cd244/Limd2/Ifng/Anxa1/Hsd11b1/Klrb1c/Cd160/Xcl1/Hopx/Cd7/Fasl/Klrd1/Il2rb/Nkg7/Cxcr3/Cxcr6 |
| TCR signaling | Ptpn6/Nfatc1/Vav3/Mapk11/Vav1/Cd3g/Cd3e/Pik3r1/Lat/Pik3cd/Itk/Lck/Lcp2/Pdcd1/Prkcq/Cd28/Cd3d/Ifng/Fyn/Cd40lg/Zap70/Il4/Icos |
| NKT activation | Il2/Il12rb2/Ccl4/Ccr4/Csf2/Ccr5/Il12rb1/Ifngr1/Cd28/Ifng/Cd40lg/Il4/Il18r1/Cxcr3 |
Table S1 (related to Figure 3, 4, 6). Leading edge genes of GSEA analysis.
